# Multifunctional Peptide-Based Biohybrid for Targeted Reduction of Metastatic Breast Carcinoma-Associated Osteolysis

**DOI:** 10.3390/jfb16110399

**Published:** 2025-10-25

**Authors:** Nicole Stadler, Bingjie Gao, Maria Jose Silva, Joscha Borho, Eva Haunschild, Kaloian Koynov, Melanie Haffner-Luntzer, Anita Ignatius, Gilbert Weidinger, Seah Ling Kuan, Tanja Weil, Holger Barth

**Affiliations:** 1 Institute of Experimental and Clinical Pharmacology, Toxicology and Pharmacology of Natural Products, University of Ulm Medical Center, Albert-Einstein-Allee 11, 89081 Ulm, Germanyjoscha.borho@uni-ulm.de (J.B.); eva.haunschild@uni-ulm.de (E.H.); 2 Max-Planck Institute for Polymer Research, Ackermannweg 10, 55128 Mainz, Germany; gaob@mpip-mainz.mpg.de (B.G.); silvam@mpip-mainz.mpg.de (M.J.S.); koynovk@mpip-mainz.mpg.de (K.K.);; 3 Institute of Orthopaedic Research and Biomechanics, Centre of Musculoskeletal Research, Ulm University Medical Center, Helmholtzstraße 14, 89081 Ulm, Germany; melanie.haffner-luntzer@uni-ulm.de (M.H.-L.); anita.ignatius@uni-ulm.de (A.I.); 4 Institute of Biochemistry and Molecular Biology, Ulm University, Albert-Einstein-Allee 11, 89081 Ulm, Germany; gilbert.weidinger@uni-ulm.de

**Keywords:** metastatic breast cancer, osteoclast, osteolysis, targeted multifunctional nanocarrier, CXCR4, EPI-X4, C3bot, Rho, avidin/biotin

## Abstract

Metastatic breast carcinoma (BC) cells are prone to spreading in the bone microenvironment, leading to a vicious cycle between local osteoclast-mediated osteolysis and tumor progression. Therefore, the targeted pharmacological down-modulation of BC cell proliferation as well as osteoclast differentiation and hyperactivity might represent a promising treatment option. We developed a multifunctional peptide nanocarrier combining bioactive EPI-X4 peptides and the Rho-inhibiting C3bot enzyme from *Clostridium botulinum*. C3bot is preferentially internalized into the cytosol of monocytic cells, including osteoclasts, where it inhibits Rho-mediated signal transduction. However, Rho-mediated cellular processes like migration and cell division can also be inhibited in non-monocytic cells if C3bot is delivered into their cytosol by a nanocarrier. To accomplish this, we designed a supramolecular transporter where one molecule of biotinylated C3bot and three biotinylated entities of the human EPI-X4 peptide-derived CXCR4 antagonist JM173 are assembled on avidin as a central platform. This modular transport system (JM173)_3_-Avi-C3 down-modulated osteoclast formation and hyperactivity and delivered the therapeutic cargo C3bot successfully into the cytosol of breast cancer cells, where it inhibited Rho.

## 1. Introduction

According to the World Health Organization, breast carcinoma (BC) is the most common cancer in women and the second-most diagnosed cancer worldwide [1]. It is associated with high mortality, especially when metastases emerge in distant organs. The most common site of systemic metastasis in breast cancer is the skeleton, leading to increased morbidity, e.g., due to pathological fractures or spinal cord compression [2,3]. In the bone niche, BC cells promote the differentiation of precursor cells to osteoclasts and enhance their activity, leading to local bone degradation. The release of minerals and nutrients from the bone in turn accelerates tumor growth and survival, creating a vicious cycle between tumor growth and osteolysis [4]. This is, in particular, a problem for triple-negative BC (TNBC), a particularly aggressive form of BC, where the cancer cells lack receptors for estrogen, progesterone, and HER2, which limits the treatment options compared to other breast cancer types [5]. Therefore, nanomaterials are being explored as a promising avenue for treating TNBC, as they offer potential advantages in drug delivery, targeting, and overcoming drug resistance, which are major challenges in TNBC treatment [5,6].

**Figure 1:**
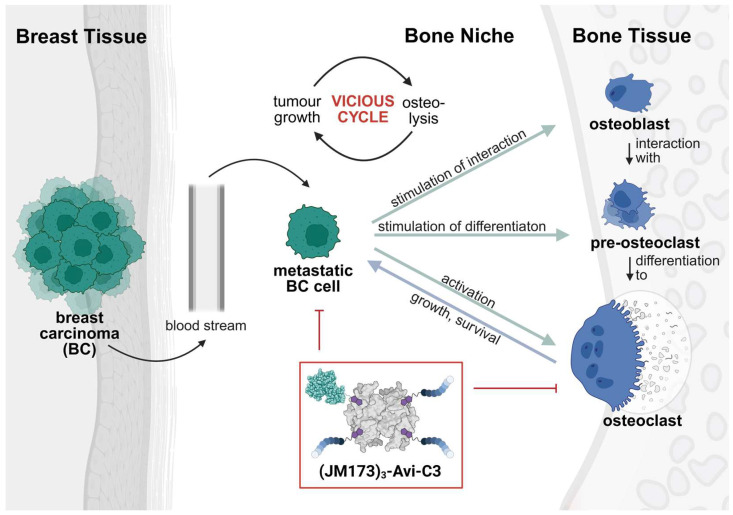
Vicious cycle between tumor growth and osteolysis in metastatic breast carcinoma (BC). In the bone niche, BC cells interact with osteoblasts to stimulate them for interaction with pre-osteoclasts, leading to intensified differentiation of the pre-osteoclasts into active osteoclasts. In addition, already existing osteoclasts are activated, promoting an overall increased degradation of bone tissue. The nutrients released from the bone are used by the metastatic BC cells and accelerate their growth and survival. The modular nanocarrier (JM173)_3_-Avi-C3 can be used to down-modulate osteoclast formation and hyperactivity and to deliver C3 toxin into the cytosol of breast cancer cells to inhibit Rho-signaling.

Regarding the vicious cycle between tumor growth and osteolysis in metastatic BC, the targeted pharmacological down-modulation of both BC cell growth and proliferation as well as osteoclast differentiation and hyperactivity by a single compound provides a promising treatment strategy. For this purpose, we used an established system for creating a multifunctional nanocarrier based on avidin/biotin technology (Figure 1) [7]. We combined the unique abilities of EPI-X4 peptides targeting the CXCR4 receptor on BC cells [8] and of the enzyme C3bot1 from the bacterium *Clostridium* (*C*.) *botulinum* (abbreviated as C3), which specifically inhibits Rho, a master-regulator of the actin cytoskeleton that is crucial for both tumor growth and osteoclast formation and activity [9,10,11].

*C. botulinum* C3 toxin is preferentially taken up into the cytosol of monocytic cells, including osteoclasts, where it specifically mono-ADP-ribosylates the GTP-binding proteins Rho A, -B, and -C [12]. This inhibits Rho-mediated signal transduction and thereby reduces Rho-/actin-mediated processes, including differentiation of osteoclasts and their osteolytic activity [10,13]. When C3 toxin is redirected to non-monocytic BC cells using artificial transporters, it can also down-modulate their Rho-/actin-mediated processes such as cell migration and division [14]. Thus, C3 could interfere on both sides of the vicious cycle and influence BC cell progression and BC-associated osteolysis if transported into the cytosol of BC cells. The redirection of C3 toxin towards BC cells is accomplished using small derivatives of the peptide JM173, which is derived from EPI-X4, an inhibitor of CXCR4 generated from human serum albumin. CXCR4 is a chemokine receptor, highly overexpressed on BC cells, where it plays a major role in metastasis and the crosstalk between BC cells and their microenvironment [15,16]. The CXCR4-targeting peptide JM173 is an improved EPI-X4 derivative with increased plasma stability and CXCR4 specificity [17]. By site-selective bioconjugation of the active components C3 toxin and JM173, a supramolecular modular nanocarrier system based on avidin (Avi) as a central platform was developed. Therefore, three entities of JM173 as well as one unit of C3 enzyme were biotinylated and assembled on avidin, creating the multifunctional peptide nanocarrier (JM173)_3_-Avi-C3 (Figure 1).

(JM173)_3_-Avi-C3 was characterized in vitro in cell-based cancer models including BC cells and in osteoclasts. Flow cytometry and fluorescence microscopy analysis revealed that JM173 mediated the specific targeting of CXCR4-overexpressing cancer cells and the cellular uptake and subsequent delivery of active C3 Rho-inhibiting enzyme into their cytosol. The latter was confirmed by biochemical analysis of the ADP-ribosylation status of Rho from cancer cells incubated in the presence of (JM173)_3_-Avi-C3. Moreover, in vitro (JM173)_3_-Avi-C3 significantly reduced both the formation of differentiated osteoclast-like cells from monocytic RAW264.7 cells and the resorption of a bone-like matrix by already differentiated osteoclast-like cells. Toxicity studies with zebrafish embryos revealed negligible toxicity of the transporter and the BC cell-targeting unit (JM173)_3_-Avi.

In conclusion, the multifunctional bioconjugate (JM173)_3_-Avi-C3 should target osteoclasts as well as metastatic BC cells in the bone niche and holds potential to reduce bone degradation in the context of BC metastasis.

## 2. Materials and Methods

*Expression and purification of recombinant GST-^Cys^C3bot1*. *Escherichia (E.) coli* BL21 (Novagene Madison, WI, USA) cells were transformed with the plasmid via heat shock. A single colony was inoculated in 5 mL LB-medium (1% tryptone, 0.5% yeast extract, 1% NaCl, 100 µg/mL ampicillin) and cultured in a shaking incubator (Benchmark Scientific, Sayreville, NJ, USA) for 5 h at 37 °C and 180 rpm. This preculture was used for inoculation of 150 mL overnight preculture in LB medium. From this second preculture, 140 mL were used to inoculate 4 L of the main culture in LB medium which grew to an OD_600_ of 0.6 to 0.8 at 37 °C and 180 rpm. After induction of protein expression with 0.5 mM isopropyl-β-D-1-thiogalactopyranoside (IPTG, Carl Roth, Karlsruhe, Germany), the main culture was incubated overnight (~18 h) at 29 °C and 180 rpm. The *E. coli* cells were harvested by centrifugation at 5500 rcf and 4 °C for 10 min and resuspended in 40 mL lysis buffer (10 mM NaCl, 20 mM Tris, 1% Triton X-100, 1% PMSF). Cell lysis was performed with EmulsiFlex-C3 homogenizer (Avestin Inc., Ottawa, Canada) at 1500 bar. Insoluble fragments were removed by centrifugation at 13,000 rcf at 4 °C for 30 min. The supernatant was filtered with 0.45 µm and 0.2 µm syringe filters and incubated overnight at 4 °C with 1.2 mL Protino Glutathione Agarose 4B-beads (Macherey-Nagel, Düren, Germany) equilibrated in PBS (137 mM NaCl, 2.7 mM KCl, 8 mM Na_2_HPO_4_, and 1.8 mM KH_2_PO_4_; pH 7.4). The beads were washed twice with washing buffer (150 mM NaCl, 20 mM Tris HCl; pH 7,4) and once with PBS by centrifugation at 4 °C and 3000 rcf for 5 min. To elute ^Cys^C3bot1, the GST-tag was cleaved by 30 NIH units of thrombin (Amersham Biosciences, Little Chalfont, UK) per liter of main culture for 1 h at room temperature. The beads were removed by centrifugation at 10,000 rcf for 30 s at 4 °C and the supernatant was transferred onto 45 µL Benzamidine-Sepharose 6B-beads (GE Healthcare, Chicago, IL, USA) and incubated at room temperature for 10 min to deplete the thrombin. The benzamidine beads were removed by 30 s centrifugation at 10,000 rcf and 4 °C. Protein concentration was determined against a BSA standard with densitometric analysis after SDS-PAGE with subsequent Coomassie staining. The ^Cys^C3bot1 enzyme was then further purified by ÄKTA pureTM chromatography system (Cytiva, Marlborough, MA, USA) using a Superdex™ 75 Increase 10/300 GL column (Cytiva, Marlborough, MA, USA) and eluted with 50 mM phosphate buffer (PB, pH 7.4) at 0.5 mL/min flow rate.

*Biotinylation of JM173*. JM173 (1.0 mg, 0.94 µmol, 1.0 equiv) was dissolved in 50 mM PB (pH 7.4) to a final concentration of 2 mg/mL. Biotin-PEG_11_-maleimide (1.0 mg, 1.13 µmol, 1.2 equiv) was dissolved in dimethylformamide (DMF) at a concentration of 10 mg/mL. The two solutions were mixed and shaken overnight at room temperature to allow for conjugation. The reaction mixture was purified by HPLC using an XDB-C18 column with a linear gradient from 5% to 100% solvent B over 19 min, followed by isocratic elution at 100% B for 3 min. After lyophilization, 1.29 mg of the product was obtained (69% yield) and characterized using mass spectrometry. Liquid chromatography-mass spectrometry was performed using Shimadzu LC-MS 2020 (Shimadzu Corporation, Kyoto, Japan), with Phenomenex Kinetex 2.6 μm EVO C18 100 Å LC 50 × 2.1 mm as the column, water, and acetonitrile with 0.1% formic acid as mobile phase. Matrix-assisted laser desorption ionization–time of flight mass spectrometry (MALDI-ToF-MS) was performed using a ToF MS rapifleX (Bruker Corporation, Ettlingen, BW, Germany). LC-MS: Tr: 3.9 min, m/z: 992 [M + 2H]^2+^. Maldi-Tof MS (CHCA): m/z: 1983 [M + H]^+^ (calcd. mass: 1982 formula: C_88_H_152_N_22_O_25_S_2_).

*Biotinylation of ^Cys^C3bot1*. The ^Cys^C3bot1 biotinylation was performed according to the previously reported literature [18]. The concentration of B-C3 was determined using Pierce Micro BCA™ Protein Assay Kit (Thermo Fisher Scientific Inc., Waltham, MA, USA), with BSA as reference at A562 nm, and the degree of biotinylation was quantified by QuantTag™ Biotin Kit (Vector Laboratories, Inc., Newark, CA, USA) at A535 nm, following manufacturers’ instructions.

*4’-hydroxyazobenzene-2-carboxylic acid (HABA)-Assay*. Avidin (Avi) and B-JM173 (0–6 equivalents) were dissolved in 25 mM HEPES buffer (pH 7.4) with a final concentration of 1 mg/mL. An amount of 70 μL of the respective protein–ligand solutions were mixed with 3.5 μL of HABA solution (1.0 mg/mL in DMSO). All mixtures were incubated at room temperature (RT) for 15 min, then transferred to a UV-Star^®^ flat-bottom 384-well plate. The absorbance at 500 nm was recorded using Tecan Spark 20M microplate reader with three technical replicates.

*Assembly of B-JM173 and B-C3 on the supramolecular platform*. Avidin was labeled with BODIPY using a previously reported labeling protocol [18]. For assembly, 10 µg of the BODIPY-labeled platform avidin (^BDP^Avi) was combined with biotinylated ^Cys^C3bot1 (B-C3) in a stoichiometric molar ratio of 1:1 (Avi to B-C3) in 50 mM HEPES buffer (Carl Roth, Karlsruhe, Germany; pH 7.4) and incubated for 45 min at 1000 rpm and room temperature. Biotinylated JM173 peptide (B-JM173) was added in a stoichiometric molar ratio of 1:1:3 (Avi:B-C3:B-JM173). B-JM173 was incubated with the pre-assembled Avi-C3 complex for 15 min under the same conditions as above. For assembly of the control constructs (JM173)_3_-Avi and Avi-C3, the respective missing component was replaced by addition of 50 mM HEPES buffer. Unbound B-JM173 was depleted by ultrafiltration in Vivaspin 500 tubes with a molecular weight cut off of 30 kDa (Sartorius, Göttingen, Germany), and the buffer system was changed to 25 mM HEPES. The concentrations of BODIPY-labeled constructs were determined by linear calibration against the respective BODIPY-labeled platform Avi at λ_abs_ = 495 nm via Nanodrop (Thermo Fisher Scientific, Waltham, MA, USA). These constructs are used for all subsequent investigations.

*Fluorescence correlation spectroscopy (FCS)*. FCS experiments were performed with a confocal microscope (LSM 880, Carl Zeiss, Jena, Germany) equipped with a C-Apochromat 40 ×/1.2 W (Carl, Zeiss, Jena, Germany) water immersion objective. An Argon laser (λ = 488 nm) fiber coupled to the LSM 880 was used for the excitation. The emission light in the spectral range 500–600 nm was detected using a spectral detection unit (Quasar, Carl Zeiss) operating in photon counting mode. For each sample, 300 μL of the studied solution was added to an 8-well polystyrene-chambered cover glass (Nunc Lab-Tek, Thermo Fisher Scientific, Waltham, MA), and series of 20 measurements, 10 sec each, were performed at room temperature (23 °C). The obtained autocorrelation curves were fitted with an analytical model function using one or two diffusing components [19]. A dye with a known diffusion coefficient, i.e., Alexa 488 was used to calibrate the confocal observation volume and thus obtain quantitative data for the hydrodynamic radius of the studied fluorescent species.

*Cell culture*. For cultivation of 4T1 cells, A431 cells, SupT1 cells, and SupT1ΔCXCR4 cells RPMI medium (Gibco-Life Technologies, Carlsbad, CA, USA) supplemented with 10% fetal bovine serum (Gibco-Life Technologies, Carlsbad, CA, USA) and 100 U/mL penicillin-streptomycin (Gibco-Life Technologies, Carlsbad, CA, USA) was used. In addition, 4T1 cell culture medium was supplemented with 1% sodium pyruvate (Gibco-Life Technologies, Carlsbad, CA, USA), 10 mM HEPES (Sigma-Aldrich, St. Louis, MO, USA), and 4.5 g/L Glucose (450 g/L stock, 0.2 µm sterile filtered, Carl Roth, Karlsruhe, Germany). The culture medium of SupT1 cells and SupT1ΔCXCR4 cells was additionally supplemented with 10 mM HEPES and 2 mM L-Glutamine (PAN-BIOTECH, Aidenbach, Germany). All cell lines were cultured at 37 °C, 5% CO_2_ with constant humidity and subcultivated every 3 to 4 days with split ratios of 1:8 to 1:20 (4T1 cells), 1:4 to 1:5 (A431 cells), and 1:3 to 1:10 (SupT1 cells and SupT1ΔCXCR4 cells). The adherent cell lines 4T1 and A431 were trypsinized (PAN-BIOTECH, Aidenbach, Germany) for splitting. RAW 264.7 cells were cultured in alpha-MEM medium (Biochrom GmbH, Berlin, Germany) supplemented with 10% fetal calf serum, 100 U/mL penicillin-streptomycin and 2 mM L-Glutamine. For differentiation, 25 ng/mL recombinant human (rh) RANKL and 10 ng/mL rhM-CSF (R&D Systems Minneapolis, MN, USA) were used. The cells were cultured at 37 °C, 5% CO_2_, and constant humidity and split at 80% confluency by adding accutase (Gibco-Life Technologies, Carlsbad, CA, USA).

*Toxicity studies*. 4T1 cells in 96-well microtiter plates were treated as indicated. After the given incubation period, 10 µL CellTiter 96^®^ AQ_ueous_ One solution containing 3-(4,5-dimethylthiazol-2-yl)-5-(3-carboxymethoxyphenyl)-2-(4-sulfophenyl)-2H-tetrazolium (MTS) were added, and cells were further incubated for 1 h. Absorbance was measured at 492 nm in a plate reader. Additionally, the toxicity of (JM173)_3_-Avi, Avi, and B-JM173 was tested in an in vivo zebrafish embryo model. To this end, dechorionated 24 h post-fertilization embryos were exposed to the substances for 24 h. Exposure was performed in 96-well microtiter plates with three embryos per well and 10 wells (= 30 embryos) per condition. The entire test was performed twice; that is, 60 embryos were assessed per condition. Cytotoxicity, cardiotoxicity, and developmental toxicity were assessed by brightfield stereomicroscopy, while neurotoxicity was tested by a touch response assay. The embryos were categorized via a standardized scoring system (Table S1) and finally assigned to one of the groups: wildtype, sublethal phenotype, or lethal phenotype.

*CXCR4 expression level.* Adherent cell lines (4T1, A431, MDA-MB231) were mechanically detached from the culture dish with a cell scrapper. Per approach, 200 µL cell suspension with a cell number of 1.0 × 10^6^ cells/mL were prepared in PBS^++^ (173 mM NaCl, 2.7 mM KCl, 8 mM Na_2_HPO_4_, 1.8 mM KH_2_PO_4_, 1 mM CaCl_2_, 0.5 mM MgCl_2_ in H_2_O) and incubated on ice for 10 min to stop unspecific endocytotic processes. For detection of CXCR4 expression levels, the indicated volumes of PE rat Anti-Human CD184 clone 1D9 (Becton, Dickinson and Company, Franklin Lakes, NJ, USA) or PE Rat IgG2a κ Isotype Control (Becton, Dickinson and Company, Franklin Lakes, NJ, USA) were added and incubated for 60 min on ice. Subsequently, the cells were washed twice with ice-cold PBS^++^ by centrifugation at 500 rcf and 4 °C for 3 min. The cells were resuspended in 200 µL PBS^++^ before fluorescence was analyzed by BD FACSCelesta™ Cell Analyzer (Becton, Dickinson and Company, Franklin Lakes, NJ, USA). Data were analyzed with Flowing Software 2.5.1 (Turku Bioscience, Turku, Finland).

*Cell binding and competition*. The cells were prepared as described in the chapter “CXCR4 expression level” before treatment. Subsequently, the cells were treated for 30 min on ice with 250 nM (JM173)_3_-^BDP^Avi-C3, (JM173)_3_-^BDP^Avi, ^BDP^Avi-C3 or ^BDP^Avi, respectively. For competition assays, free non-biotinylated JM173 peptide was added simultaneously to this reaction in the indicated concentrations. Washing of the cells, measurement via flow cytometry, and data analysis was performed as described in the chapter “CXCR4 expression level”.

*Phase-contrast microscopy*. Cells grown in microtiter plates were treated as indicated, and cell morphology was recorded by phase contrast microscopy (Leica microsystems, Wetzlar, Germany). In the figures, representative images with scale bars are shown.

*Fluorescence microscopy*. Cells were seeded in an 8-well µ-slide (ibidi GmbH, Gräfelfing, Germany), treated with the indicated proteins and concentrations and incubated overnight at 37 °C, 5% CO_2_ with constant humidity. The cells were washed twice with PBS and fixed with 4% paraformaldehyde (Merck KGaA, Darmstadt, Germany) in PBS for 20 min at room temperature. After three washing steps with PBS, the actin cytoskeleton was stained by incubating with SiR-actin (Spirochrome, Thurgau, Switzerland) in a 1:2000 dilution in PBS for 45 min at room temperature. The cells were washed three times with PBS, and the nucleus was stained with Invitrogen^TM^ Hoechst 33342 (Thermo Fisher Scientific, Waltham, MA, USA) 1:5000 diluted in PBS for 10 min at room temperature. The washed cells were stored in 0.01% NaN_3_ (Carl Roth, Karlsruhe, Germany) in PBS at 4 °C in the dark. Images were taken with Keyence BZ-X810 fluorescence microscope using DAPI, GFP, and Cy5 filter (Keyence Corporation, Osaka, Japan).

*SDS-PAGE and Western blot*. SDS-PAGE was performed by using a 12.5% acrylamide gel for protein separation. Subsequently, the gel was either stained with Coomassie Brilliant Blue R250 (SERVA Electrophoresis GmbH, Heidelberg, Germany) or the proteins were transferred onto a nitrocellulose membrane by semi-dry Western blotting. To control protein transfer, Ponceau S (AppliChem GmbH, Darmstadt, Germany) staining was performed before the membrane was blocked in 5% skim milk powder solution in PBS-T (137 mM NaCl, 2.7 mM KCl, 8 mM Na_2_HPO_4_, 1.8 mM KH_2_PO_4_, 0.1% Tween20; pH 7.4) for 1 h at room temperature. Incubation and detection via antibodies were performed as indicated.

*Sequential ADP-Ribosylation*. 4T1 cells were seeded in 24-well microtiter plates 24 h prior to the indicated treatment and incubated overnight at 37 °C and 5% CO_2_. Washing twice with PBS removed residual toxin before the cells were lysed at -20 °C for at least 30 min. The cells were scratched off the plate in ADP-ribosylation buffer (20 mM Tris-HCl, 1 mM EDTA, 1 mM DTT, 5 mM MgCl_2_, cOmplete^TM^ (1:50, freshly added); pH 7.5) and mixed with 10 pmol C3lim as well as 1.5 µL fluorescein-NAD^+^ (Bio-Techne, Minneapolis, MN, USA). The sequential ADP-ribosylation reaction was performed at 37 °C for 30 min and stopped by adding Laemmli buffer (0.3 M Tris-HCl, 10% SDS, 37.5% glycerol, 0.4 mM bromophenol blue) and heat denaturation at 95 °C for 10 min. SDS-PAGE and Western blot were performed using an anti-fluorescein (FITC) antibody (clone 5D6.2, 1:500 in PBS-T, Merck KGaA, Darmstadt, Germany) to detect Rho protein that was fluorescein-labeled during the sequential ADP-ribosylation reaction. Thus, a weak signal in the Western blot corresponds to a high ADP-ribosyltransferase activity of the toxin in living cells (i.e., a strong intoxication of cells before lysis). Hsp90 was used as a loading control.

*Proliferation assay.* MDA-MB231 cells were seeded in 96-well microtiter plates (Thermo Fisher Scientific, Waltham, MA, USA) 24 h prior to treatment and incubated overnight at 37 °C and 5% CO_2_. The cells were treated with 250 nM (JM173)_3_-^BDP^Avi-C3, (JM173)_3_-^BDP^Avi, ^BDP^Avi-C3, ^BDP^Avi, C3bot1 or 750 nM JM173, respectively. Live-cell imaging was performed with the Incucyte^®^ SX5 (Sartorius, Göttingen, Germany) using the “Adherent Cell-by-Cell” scan type at 10-fold magnification for 5 days. Data analysis was performed with the software Incucyte^®^ 2024A (Sartorius, Göttingen, Germany).

*Effect of (JM173)_3_-^BDP^Avi-C3-treatment on osteoclast formation*. To assess the influence of the multifunctional nanocarrier on osteoclast-formation, 37.5 nM, 150 nM or 250 nM (JM173)_3_-^BDP^Avi-C3, (JM173)_3_-^BDP^Avi, ^BDP^Avi-C3, ^BDP^Avi or JM173 were administered to RAW 264.7 cells during their differentiation to osteoclasts from day 0 on, respectively. Osteoclastic differentiation was induced by adding 25 ng/mL recombinant human (rh) RANKL and 10 ng/mL rhM-CSF. Following one medium change on day 3, the medium was removed on day 5 to stain the differentiated osteoclasts with Acid Phosphatase, Leukocyte (TRAP) Kit according to the manufacturer (Sigma-Aldrich, St. Louis, MO, USA). The multi-nucleated TRAP-positive cells were counted as osteoclasts at 100-fold magnification with a Leica DMI6000 B microscope and a DFC420 C camera (Leica Microsystems, Mannheim, Germany).

*Effect of (JM173)_3_-^BDP^Avi-C3-treatment on osteoclast resorption activity*. The resorption activity of osteoclasts was assessed in OsteoAssay human bone plates (Lonza, Basel, Switzerland). RAW 264.7 cells were seeded with a cell number of 1*10^4^ cells per mL and 100 µL/well and treated with differentiation medium supplemented with 37.5 nM (JM173)_3_-^BDP^Avi-C3, (JM173)_3_-^BDP^Avi, ^BDP^Avi-C3, ^BDP^Avi or JM173, respectively. A medium change with all additives was performed on day 3 and day 7 after seeding. On day 10, the OsteoAssay bone plates were filled with 5% trypsin for 5 min to detach the cells and washed twice with water. Afterwards, mineralization was stained by Von Kossa staining. When imaging the plate with a Leica DMI6000 B microscope and a DFC420 C camera, resorbed areas by osteoclast-like cells were visible in transparent color, while the non-resorbed surface appeared black. The resorption area was quantified using the image processing software LASX (Leica Microsystems, Mannheim, Germany).

## 3. Results

A supramolecular peptide/protein-based transporter was generated harboring one molecule of biotinylated C3bot1 (B-C3) and three biotinylated entities of the human EPI-X4 peptide-derived CXCR4 antagonist JM173 (B-JM173) on a biotin-binding tetrameric avidin platform. The seven amino acids long JM173 peptide should mediate the targeted delivery of the therapeutic cargo molecule C3bot1 toxin (indicated in this context as C3) into the cytosol of CXCR4 overexpressing breast carcinoma (BC) cells. Thus, the modular transport system (JM173)_3_-Avi-C3 should not only downmodulate osteoclast formation and hyperactivity but also the growth of BC cells in the bone niche.

**Figure 2:**
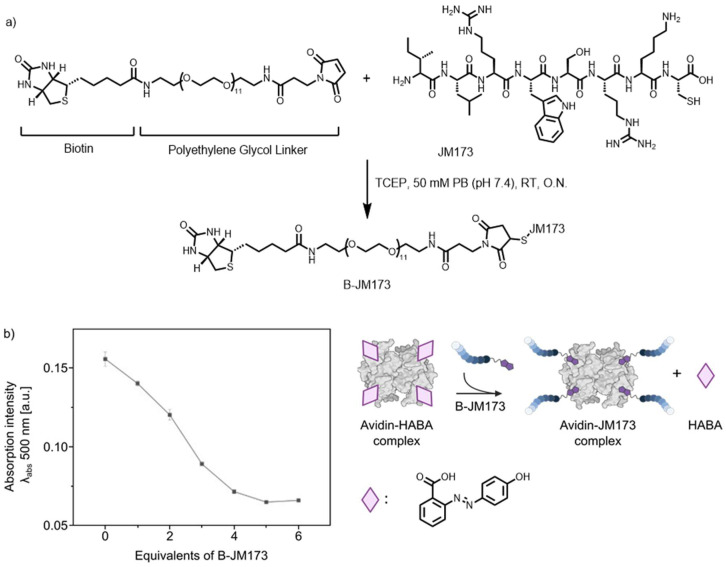
Synthesis of biotinylated JM173 peptide (B-JM173) and optimization of the binding ratio between B-JM173 and avidin. (**a**) Synthetic route for biotinylation of JM173. B-JM173 was obtained in 69% yield after HPLC purification and characterized using MALDI-Tof MS (CHCA): m/z (observed), 1983 [M + H]^+^, 2005 [M + Na]^+^, (calcd. mass, 1982 formula: C_88_H_152_N_22_O_25_S_2_). (**b**) Competitive binding of 4’-hydroxyazobenzene-2-carboxylic acid (HABA) and biotin to avidin to determine the amount of B-JM73 required to saturate the tetrameric biotin-binding protein. Four molar equivalents of B-JM173 saturated the corresponding binding sites; values are given as mean ± SD (n = three technical triplicates).

To generate this transporter, JM173 was functionalized with a bifunctional maleimide-biotin containing stable polyethylene glycol linker to improve water solubility (Figure 2a). The biotinylated JM173 peptide (B-JM173) was purified by high-performance liquid chromatography (HPLC) and characterized using LC-MS and MALDI-ToF analysis (Figure S1). To calculate the ratio of B-JM173 that can be bound to the tetrameric biotin-binding Avi, a competitive binding assay with 4’-hydroxyazobenzene-2-carboxylic acid (HABA) was performed (Figure 2b). Due to the higher affinity of biotin for the platform, the anionic dye HABA is displaced by B-JM173 resulting in a proportional decrease in absorption at 500 nm and indicating a controlled binding of B-JM173 to the platform until saturation of the binding pocket is achieved, as indicated by a plateau. It was determined that each binding pocket in Avi required one molar equivalent of B-JM173.

The second effector of the supramolecular complex, ^Cys^C3bot1, was also biotinylated using a bifunctional biotin-maleimide conjugate with a pH-sensitive hydrazone linker according to a previously published procedure [18] (Figure S2a). MALDI-ToF analysis showed successful conjugation to the maleimide reagent (Figure S2c). A Western blot detected with Strep-POD conjugate showed that the linker is structurally intact and contains biotin after modification. In addition, it confirms that there was no free biotin linker in the system after biotinylation of C3 toxin (Figure S2b). The degree of biotinylation ranged from 40 to 89%, as determined using microbicinchoninic acid (µBCA) assay and biotin quantitation kit.

**Figure 3:**
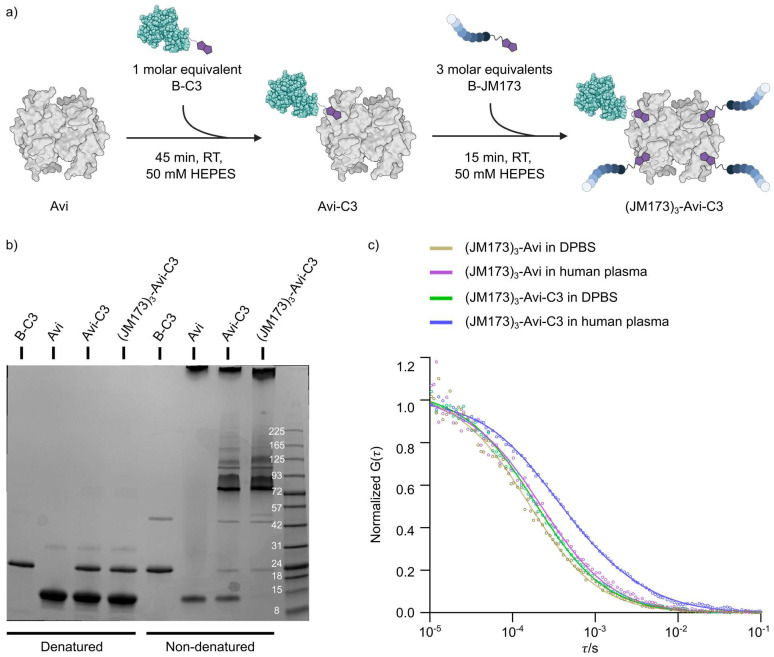
Assembly of (JM173)_3_-Avi-C3 and its stability in DPBS and human plasma. (**a**) Assembly of B-C3, B-JM173 and avidin by a stochiometric control-based reaction. (**b**) SDS-PAGE analysis of the assembled complex under denaturing and non-denaturing conditions. Weak B-C3 bands under non-denaturing conditions indicate successful assembly of (JM173)_3_-Avi-C3. (**c**) Fluorescence correlation spectroscopy (FCS) analysis of (JM173)_3_-Avi-C3 and (JM173)_3_-Avi in DPBS and human plasma. The average hydrodynamic radii of (JM173)_3_-Avi, and (JM173)_3_-Avi-C3 in DPBS were 3.6 nm and 4.3 nm, while in human plasma they were 3.5 nm and 4.3 nm (~80%), respectively. Additionally, particles with a hydrodynamic radius of around 20 nm were observed for (JM173)3-Avi-C3 in plasma. Measurements were carried out using BODIPY-labeled constructs.

The controlled assembly of B-C3, B-JM173 and avidin was accomplished by stoichiometric control in the molar ratio of 1:1:3 (Avi:B-C3:B-JM173) (Figure 3a). SDS-PAGE analysis of the different supramolecular complexes under denatured and non-denatured conditions showed that almost all B-C3 was involved in the assembly process using the avidin platform (Figure 3b). For all subsequent studies, we assembled (JM173)_3_-Avi and (JM173)_3_-Avi-C3 using BODIPY-labeled Avi. The stability of those biohybrids was first investigated in cell culture medium and in human plasma. Therefore, fluorescence correlation spectroscopy was used to determine the average hydrodynamic radii of (JM173)_3_-Avi and (JM173)_3_-Avi-C3 in DPBS (3.6 nm and 4.3 nm, respectively) and in human plasma (3.5 nm and 4.3 nm, respectively) (Figure 3c). The size of (JM173)_3_-Avi in human plasma did not change compared to that in DPBS, indicating that it was not degraded. In addition, the fitting of the FCS curves measured in human plasma showed that most of the (JM173)_3_-Avi-C3 (~80%) remained as a heterodimeric protein particle with a hydrodynamic radius of 4.3 nm. Some particles with an average hydrodynamic radius of about 20 nm appeared, in contrast to the observation in DPBS, possibly due to interactions with human plasma components. However, no larger aggregates were formed in human plasma, which might result in loss of bioactivity of the supramolecular complex.

**Figure 4:**
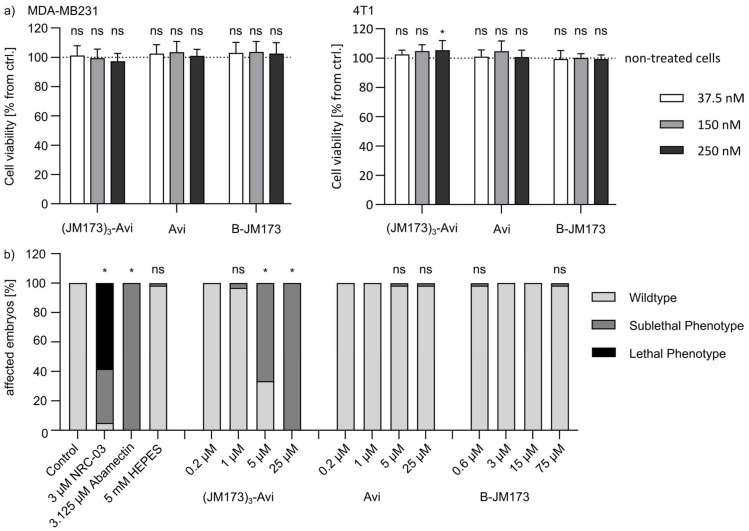
Toxicity studies of the assembly platform avidin and the CXCR4-targeting entity JM173. (**a**) MTS assay with 4T1 cells treated with 37.5 nM, 150 nM or 250 nM (JM173)3-Avi or Avi for 72 h. Detection of the relative viability (% from non-treated cells) of the 4T1 cells. The values are given as mean ± SD of triplicates from three individual experiments. Statistical analysis was performed by using non-parametric one-way ANOVA (MDA-MB231 cells: *F*(6,96) = 0.293, *p* = 0.9391, 4T1 cells: *F*(6,96) = 0.024, *p* > 0.999) in combination with Dunnett’s correction for multiple comparison against the non-treated cells (ns *p* > 0.05, * *p* < 0.05). (**b**) In vivo toxicity study in zebrafish embryos which were treated with the indicated concentration of (JM173)3 Avi, Avi or B-JM173 for 24 h. NRC-03 served as a positive control for cytotoxic effects, abamectin as a positive control for neurotoxicity, and HEPES as a solvent control. The embryos were categorized via a standardized scoring system (Table S1) and assigned into the groups “wildtype” (bright gray), “sublethal phenotype” (dark gray) or “lethal phenotype”, black). Results shown are the combined data from two independent tests of 30 embryos each per group; that is, for 60 embryos per group. Statistical analyses were performed using the Chi-Square test (n.s. *p* > 0.05, * *p* < 0.001).

The successful stability tests were followed by investigating the effect of the cell-targeting unit (JM173)_3_-Avi on cell viability. Therefore, an MTS assay was performed using the mouse breast carcinoma model cell line 4T1 and the mammary gland adenocarcinoma cells MDA-MB231. Increasing concentrations of (JM173)_3_-Avi were incubated with the 4T1 cells for up to 72 h without showing any negative effects on cell viability (Figure 4a). Based on these results, an in vivo toxicity study on zebrafish embryos was applied. A standardized scoring system (Table S1) was used to evaluate embryonic mortality after 24 h of exposure to (JM173)_3_-Avi, Avi or B-JM173, as well as sublethal cytotoxicity, developmental toxicity, and toxicity affecting specific organ systems, particularly cardiotoxicity and neurotoxicity. The transporter (JM173)_3_-Avi showed weak toxic effects in concentrations higher than 5 µM. This toxicity could be classified as very mild, sublethal cell lysis that did not prevent further development and survival of the embryos (Figure 4b). In conclusion, the transporter and cell-targeting unit (JM173)_3_-Avi did not show cytotoxic effects in concentrations used for further studies.

**Figure 5:**
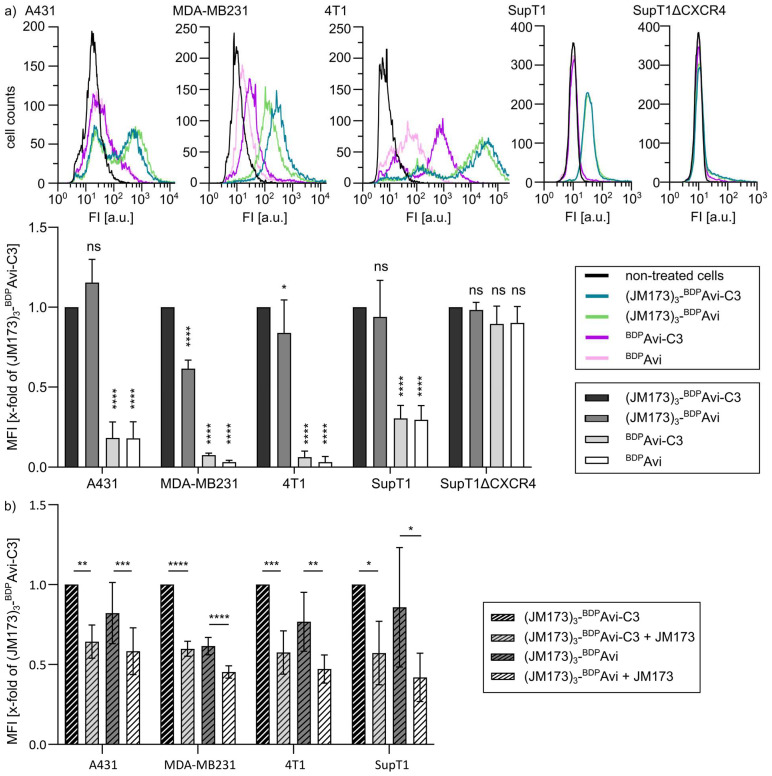
Binding of (JM173)_3_-^BDP^Avi-C3 and control constructs to different cell lines in absence (**a**) or presence (**b**) of free JM173 peptide. (**a**) The cell lines A431, MDA-MB231, 4T1, SupT1, and SupT1 cells with CXCR4 knockout (SupT1ΔCXCR4) were treated on ice for 30 min with 250 nM (JM173)_3_-^BDP^Avi-C3, (JM173)_3_-^BDP^Avi, ^BDP^Avi-C3 or ^BDP^Avi, respectively, or left untreated as control. Cells were washed twice and applied to flow cytometric analysis. The representative histograms display single-cell events plotted against cellular fluorescence (log (FI)). The quantitative analysis shows the results of three independent experiments. MFI-values were normalized to (JM173)_3_-^BDP^Avi-C3 and represent the x-fold change in median fluorescence intensity (MFI) compared to (JM173)_3_-^BDP^Avi-C3 ± SD. Significance was tested against (JM173)_3_-^BDP^Avi-C3 using one-way ANOVA (A431: *F*(3,8) = 78.89, *p* < 0.0001; MDA-MB231: *F*(3,20) = 1617, *p* < 0.0001; 4T1: *F*(3,24) = 159, *p* < 0.0001; SupT1: *F*(3,12) = 35.93, *p* < 0.0001; SupT1ΔCXCR4: *F*(3,12) = 1.871, *p* = 0.188) combined with Dunn’s multiple comparison test (ns *p* > 0.05, * *p* < 0.05, **** *p* < 0.0001). (**b**) Competition assay with free JM173 added simultaneously to the treatments described in (**a**). Values represent the x-fold change in median fluorescence intensity (MFI) compared to (JM173)_3_-^BDP^Avi-C3 ± SD. The quantitative analysis shows the results of three independent experiments. Significance was tested against the respective condition without free JM173 using one-way ANOVA (A431: *F*(3,16) = 10.31, *p* = 0.0005; MDA-MB231: *F*(3,20) = 196.7, *p* < 0.0001; 4T1: *F*(3,16) = 17.98, *p* < 0.0001; SupT1: *F*(3,12) = 5.532, *p* = 0.013) combined with Dunn’s multiple comparison test (* *p* < 0.05, ** *p* < 0.01, *** *p* < 0.001, **** *p* < 0.0001).

Having confirmed the identity of the supramolecular transporter, the cellular uptake, cell-type-selectivity, and biological activity of (JM173)_3_-Avi-C3 were the investigated in cell-based models of breast cancer (BC) and osteoclasts. First, we analyzed the uptake of the transporter into cells, including BC cells. To this end, the expression of CXCR4 in these cell lines was measured with a fluorescently labeled anti-CXCR4 antibody. As shown, epidermoid carcinoma cells A431, T cell lymphoma cells SupT1, mammary gland adenocarcinoma cells MDA-MB231 and 4T1 cells, a mouse model of stage IV human breast cancer, expressed CXCR4 and were therefore proper model cell lines for further examination of the binding of (JM173)_3_-Avi-C3 and the delivery of C3 into their cytosol (Figure S3). To analyze the binding of (JM173)_3_-Avi-C3, these cell lines were incubated with fluorescently labeled (JM173)_3_-^BDP^Avi-C3 or the control constructs (JM173)_3_-^BDP^Avi, ^BDP^Avi-C3, and ^BDP^Avi, and binding of these constructs to the cells was analyzed by flow cytometry. A right-shift in fluorescence compared to non-treated cells indicates cell surface binding of fluorescently labeled JM173-containing constructs. The results revealed that binding to CXCR4-bearing cells was significantly enhanced for constructs containing the JM173 peptide as compared to constructs without the JM173 peptide. Here, enhanced binding is indicated by a right-shift in fluorescence intensity, which is directly proportional to the amount of cell-bound fluorescent construct (Figure 5a). In contrast, SupT1 cells with CXCR4 knock-out (SupT1ΔCXCR4) did not show binding of any construct to the cell surface, indicating CXCR4-specific binding of the JM173-containing constructs (Figure 5a). Moreover, co-incubation with an excess of free JM173 peptide reduced the binding of JM173-containing constructs, further corroborating the receptor-specific interaction of JM173 and CXCR4 (Figure 5b). In conclusion, these results confirm that binding of (JM173)_3_-^BDP^Avi-C3 strongly depends on JM173 and the presence of CXCR4 on the surface of target cells.

**Figure 6:**
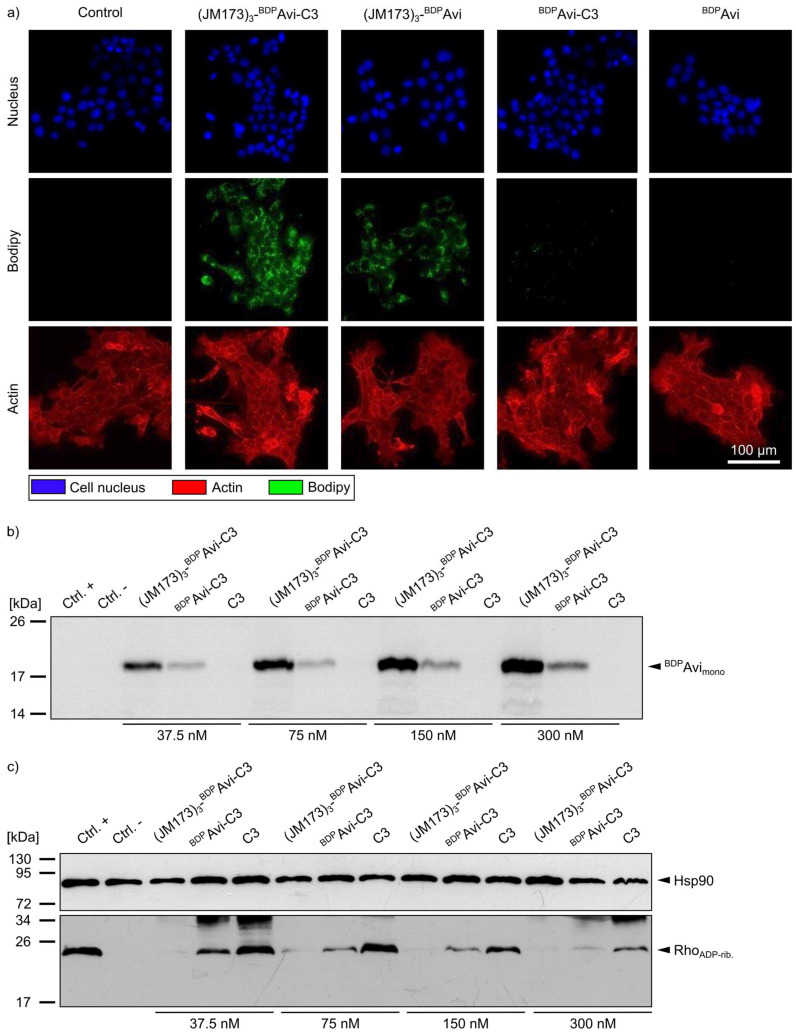
Intracellular uptake of (JM173)_3_-^BDP^Avi-C3 into 4T1 cells. (**a**) 4T1 cells were treated with 250 nM (JM173)_3_-^BDP^Avi-C3, (JM173)_3_-^BDP^Avi, ^BDP^Avi-C3 or ^BDP^Avi overnight before washing twice with PBS. The cells were fixed, and immunostaining was performed with SiR-Actin (red, actin) and Hoechst (blue, nuclei). Afterwards, BODIPY-labeled constructs (green) were detected by fluorescence microscopy. (**b**) 4T1 cells were intoxicated with the indicated concentrations of (JM173)_3_-^BDP^Avi-C3, ^BDP^Avi-C3 or C3bot1 (C3) for 8 h. The cells were washed and lysed before C3lim and NAD^+^-fluorescein were added to label non-ADP-ribosylated Rho with fluorescein (sequential ADP-ribosylation reaction). Non-intoxicated 4T1 cells that were subsequently treated with fluorescein-NAD^+^ and with (ctrl. +) or without (ctrl. −) C3lim in the sequential ADP-ribosylation reaction served as controls. After SDS-PAGE and Western blot, the membrane was imaged at 500 nm to visualize BODIPY-labeled avidin monomers (~17 kDa), indicating concentration-dependent binding and uptake of the constructs into the cells during intoxication. (**c**) The membrane from (**b**) was additionally detected for the amount of fluorescein-labeled Rho (i.e., Rho that has not been ADP-ribosylated by C3 before cell lysis) by immunoblotting, and Hsp90 served as loading control. Weak signals indicate strong intoxication in the first assay step.

Fluorescence microscopy of 4T1 cells treated with (JM173)_3_-^BDP^Avi-C3, (JM173)_3_-^BDP^Avi, ^BDP^Avi-C3, and ^BDP^Avi revealed that uptake of C3 into non-monocytic cells was only detectable for the JM173-containing modular bioconjugates (Figure 6a, green signal). Moreover, this finding was verified via immunoblotting of 4T1 cells treated with (JM173)_3_-^BDP^Avi-C3, ^BDP^Avi-C3 or C3 for 24 h. Analysis of blotted cell lysates at an excitation wavelength of 500 nm to visualize the amount of formerly internalized fluorescently labeled avidin platform confirmed the ability of JM173-containing constructs to enter the cytosol of 4T1 cells in a concentration-dependent manner (Figure 6b). These results were further confirmed by the biochemical analysis of the ADP-ribosylation status of Rho in 4T1 cells that were incubated with (JM173)_3_-^BDP^Avi-C3, ^BDP^Avi-C3 or C3, respectively. After cell lysis, the amount of non-ADP-ribosylated Rho was detected by Western blotting. Here, a strong signal indicates no or weak ADP-ribosylation of Rho in the intact cells during the incubation period, while a weak signal indicates a strong ADP-ribosylation of Rho (i.e., a strong intoxication) in these cells before lysis (Figure 6c). Whereas treatment of 4T1 cells with C3 alone led only to marginal ADP-ribosylation of Rho, almost all Rho was ADP-ribosylated in cells incubated with (JM173)_3_-^BDP^Avi-C3, indicating that active C3 was delivered into the cytosol of the cells by this construct. While our results indicate that high concentrations of ^BDP^Avi without JM173 also seem to enhance C3-uptake to a certain degree, this effect is much stronger for JM173-containing bioconjugates. Thus, (JM173)_3_-^BDP^Avi-C3 is a promising candidate to target metastatic BC cells overexpressing CXCR4.

**Figure 7:**
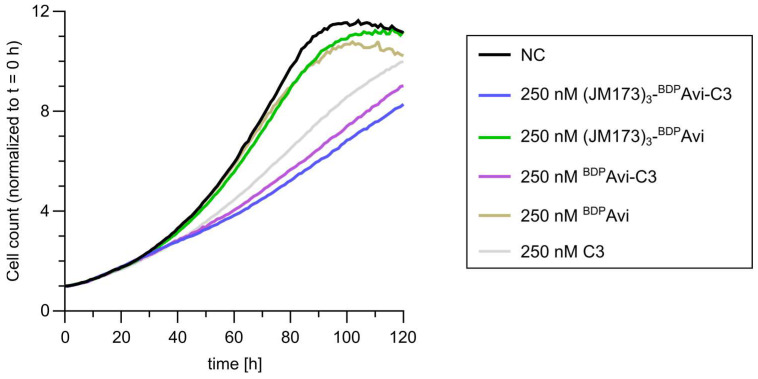
Live-cell monitoring of the proliferation of MDA-MB231 cells treated with the indicated constructs and proteins or left untreated for control (NC). The data represent the cell count per image over time normalized to the respective cell count at t = 0 h. The graph shows the mean values of three independent experiments analyzed with the Incucyte^®^ 2024A software using the adherent cell-by-cell software module.

To confirm the inhibitory effect of JM173-mediated C3 intoxication on actin-dependent cell functions like proliferation, live-cell imaging was performed. To this end, MDA-MB231 cells were treated with (JM173)_3_-^BDP^Avi-C3 and control constructs, as well as wildtype C3 toxin and free JM173 in the indicated equimolar ratios (Figure 7). This assay clearly showed that cell proliferation was more strongly inhibited by the (JM173)_3_-^BDP^Avi-C3 construct as compared to C3 alone. These results are in line with the previously shown effect of the fully assembled construct’s effect on the ribosylation status of Rho.

**Figure 8:**
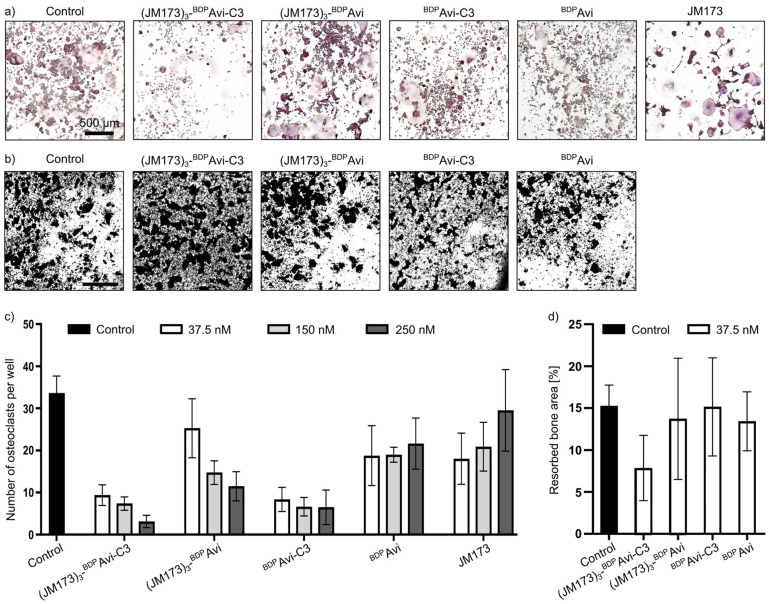
The effect of (JM173)_3_-^BDP^Avi-C3 on osteoclast formation and resorption activity. (**a**) RAW264.7 cells were differentiated with 25 ng/mL rhRANKL and 10 ng/mL rhM-CSF in absence (control) or presence of 37.5 nM of (JM173)_3_-^BDP^Avi-C3, (JM173)_3_-^BDP^Avi, ^BDP^Avi-C3, ^BDP^Avi, or JM173, respectively. On the 5th day, the multi-nuclear osteoclasts were TRAP-stained. The representative pictures show that C3-containing constructs decreased the number and size of formed osteoclasts drastically. The scale bar measures 500 µm. The experiment was performed as a biological triplicate. (**b**) RAW264.7 cells were seeded into an OsteoAssay human bone plate and differentiated with 25 ng/mL rhRANKL and 10 ng/mL rhM-CSF in the absence (control) or presence of 37.5 nM (JM173)_3_-^BDP^Avi-C3, (JM173)_3_-^BDP^Avi, ^BDP^Avi-C3, or ^BDP^Avi, respectively. On the 10th day, the cells were removed from the plate and mineralization was stained by Von Kossa staining. Non-resorbed areas appear black, while resorbed areas are transparent. All approaches were performed as a technical quadruplicate (control) or septuplicate (treatment conditions), and representative images are shown. (JM173)_3_-^BDP^Avi-C3 decreases the bone resorption activity of osteoclasts compared to the other control constructs. The scale bar measures 500 µm. (**c**) Quantitative analysis of osteoclast formation from (**a**) with treatment of 37.5 nM, 150 nM or 250 nM of the labeled substances. Data show the results of two independent experiments with a total of n = 24 (control) or n = 8 (per treatment condition). Values represent the number of osteoclasts per well ± SD. C3-containing constructs lower the number of differentiated osteoclasts in a concentration-dependent manner. All experimental groups except 250 nM JM173 showed significant reduction compared to control. (**d**) Quantitative analysis of osteoclast resorption activity from (**b**). Values represent the resorbed bone area per well in percentage ± SD.

Next, it was investigated whether (JM173)_3_-^BDP^Avi-C3 exhibited the expected inhibitory effects on osteoclast differentiation and resorption activity. As shown in Figure 8, the fully assembled complex significantly reduced the formation of differentiated osteoclast-like cells from monocytic RAW264.7 cells in vitro (Figure 8a,c) and the resorption of a human bone-like matrix by already differentiated osteoclast-like cells, as analyzed by Von Kossa staining (Figure 8b,d).

Taken all together, biotinylated JM173 peptides mediate cell type-selective delivery of the modular bioconjugate (JM173)_3_-Avi-C3 into CXCR4-expressing cancer cells and the release of the therapeutic cargo protein C3 into their cytosol to down-modulate Rho-/actin-dependent growth of cancer cells. Moreover, application of (JM173)_3_-Avi-C3 reduced osteoclast formation and bone resorption activity via C3 activity.

## 4. Discussion

A more specific targeting and combating of tumor cells and comorbidities at metastasis sites is a central goal in cancer therapy. Patients suffering from late-stage breast carcinoma (BC) often exhibit skeletal-related disorders due to the vicious cycle between BC metastases and osteoclasts in the bone niche [3,4]. In this study, we generated the modular bioconjugate (JM173)_3_-Avi-C3 for targeted down-modulation of both osteoclast formation and hyperactivity as well as growth of breast cancer cells, particularly in patients with triple-negative BC. To inhibit detrimental cellular processes in both targeted cell types, it is crucial to introduce the Rho-inhibitor C3bot into their cytosol. Prompted by our earlier approaches to generate chemically engineered supramolecular multidomain protein/peptide complexes [7,18] and a supramolecular avidin photosensitizer that targets triple negative BC [20], avidin/biotin technology was used to obtain the bioconjugate (JM173)_3_-Avi-C3. This bioconjugate allows for the simultaneous targeting of different cell types by the three biotinylated JM173 peptides, which act as selective CXCR4 receptor antagonists to target the BC cells [17], and one biotinylated C3 enzyme targeting the osteoclasts in the bone niche.

A series of in vitro experiments were performed to confirm the identity of this bioconjugate and demonstrate its pharmacological properties. Bone is a common site of CXCR4-positive cancer metastases probably attracted by the elevated concentration of the natural CXCR4 ligand SDF-1 in the bone marrow [21]. Our study showed that the presence of JM173 peptides was crucial for the selective binding of (JM173)_3_-Avi-C3 to CXCR4-overexpressing cancer cells. Since the construct is intended to be used for the local treatment of breast cancer metastases in the bone niche, potential off-target effects on the CXCR4-expressing bone marrow must be considered. However, the bone marrow is protected by the dense cortical bone, the trabecular bone, and the endosteum, separating the targeted osteoclast/osteoblast remodeling zones from the inner bone structures [22,23]. Other CXCR4 inhibitors like plerixafor or balixafortide have already been tested against several types of cancers combined with chemotherapy or cytostatic drugs for improved chemosensitivity and response rates without showing severe side effects linked to bone marrow dysregulation [24,25].

After selective binding of JM173 to the CXCR4-overexpressing breast cancer cells, (JM173)_3_-Avi-C3 is taken up into the cytosol. This could be confirmed by down-modulation of proliferation in human breast adenocarcinoma cells (MDA-MB231) and mouse model stage IV breast cancer cells (4T1) induced by inhibition of Rho/actin-signaling pathways [9]. The cell binding and translocation mechanism for C3 toxin is not yet fully understood [26,27,28]. Although it has been shown that monocytic cells (e.g., osteoclasts) are naturally capable of efficient C3 uptake, other cell types are rather poorly affected by C3 intoxication [13,29,30]. We propose that the spatial proximity of C3 to the cell membrane after JM173 binding to CXCR4 could accelerate the specific uptake of (JM173)_3_-Avi-C3 into breast cancer cells. This hypothesis is strengthened by the poor internalization of C3 toxin into 4T1 cells in the absence of JM173 as shown in the sequential ADP-ribosylation assay.

The (JM173)_3_-Avi-C3 bioconjugate did not only affect breast cancer cells but also showed a strong inhibition of osteoclast formation and bone resorption, which is most likely based on the activity of cytosolic C3. It has been reported earlier that C3 toxin is taken up into osteoclast-like cells, leading to inhibition of osteoclastogenesis and disrupting the sealing zone of osteoclasts [10,13,31]. (JM173)_3_-Avi-C3 might have several advantages and disadvantages compared to clinically available anti-osteolytic/anti-resorptive therapies like bisphosphonates, denosumab, and other indirect anti-osteolytic compounds. Bisphosphonates like zoledronic acid, alendronate, and pamidronate remain the cornerstone of clinical management, bind to hydroxyapatite in bone, and are internalized by osteoclasts during resorption. Nitrogen-containing bisphosphonates inhibit farnesyl pyrophosphate synthase in the mevalonate pathway, disrupting osteoclast function and promoting apoptosis. They have proven efficacy in reducing pathological fractures, bone pain, and hypercalcemia in cancer-related bone disease as well as in osteoporosis [32]. However, the very long-lasting mode of action of bisphosphonates can cause side-effects that could be overcome with (JM173)_3_-Avi-C3. Denosumab, a fully human monoclonal antibody targeting the receptor activator of nuclear factor κB ligand (RANKL), offers a potent and reversible alternative. By preventing RANKL from binding to its receptor RANK on osteoclast precursors, denosumab effectively suppresses osteoclastogenesis. It is administered subcutaneously and is indicated both for osteoporosis and for the prevention of skeletal-related events in metastatic cancers. Unlike bisphosphonates, denosumab is not renally cleared, making it suitable for patients with renal impairment [33,34]. A disadvantage of denosumab is its very high costs, also compared to (JM173)_3_-Avi-C3. However, it needs to be proven in clinical trials if (JM173)_3_-Avi-C3 is of similar potency to denosumab. Other therapies with indirect anti-osteolytic effects include selective estrogen receptor modulators (SERMs), calcitonin, and anabolic agents such as teriparatide, which improve bone remodeling balance [35,36]. In oncology, targeted agents that inhibit tumor-derived osteolytic cytokines (e.g., IL-6 or PTHrP) and emerging anti-sclerostin antibodies (e.g., romosozumab) further contribute to bone protection [37,38,39]. The advantages of these compounds over (JM173)_3_-Avi-C3 are their bi-directional action regarding enhancement of osteoblasts and inhibition of osteoclasts. Nevertheless, (JM173)_3_-Avi-C3 also exhibits bi-directional activities, since it is not only capable of targeting the BC cells but also comorbidities accruing due to BC cell metastases in the bone niche.

For in vivo applications, toxicity and pharmacokinetics are important issues. Toxicity studies with cultured cells and zebrafish embryos revealed negligible toxicity of the supramolecular complex (JM173)_3_-Avi. Regarding pharmacokinetics, biotin–protein bonds are susceptible to hydrolysis in human plasma [40]. By using spectroscopic measurements and defining the hydrodynamic radii of (JM173)_3_-Avi and (JM173)_3_-Avi-C3 in human plasma, we have shown that JM173-PEG_11_-maleimide and C3bot-linker-maleimide [18] were stably connected with biotin, forming a robust nanocarrier when bound to avidin. Avidin is highly positively charged and tends to exhibit weak, nonspecific, and transient electrostatic binding to negatively charged plasma components [41,42]. In the present study, only 20% of (JM173)_3_-Avi-C3 interacted with plasma proteins, leading to an increase in its hydrodynamic radius. This could be further improved by alternative platforms for binding the biotinylated peptides/proteins. Using streptavidin isolated from *Streptomyces avidinii* as well as the chemically modified avidin-variant neutravidin as platforms could improve the stability of the construct in human plasma and lower the interactions with plasma proteins [43]. In addition, streptavidin and neutravidin have been shown to induce reduced immunogenic effects compared to avidin when applied to mammals [44]. Nevertheless, even though basal human anti-avidin antibody titers can be measured in blood and are rising upon avidin treatment, no antibody-related clinical symptoms were observed in humans or animals [45].

However, there are several aspects that need to be considered before further in vivo studies with (JM173)3-Avi-C3 can be performed. Bioavailability, potential metabolism, and degradation under physiological conditions in the relevant tissues are strongly related to the mode of administration, i.e., systemic or locally into the bone niche. One concept for local application is the use of hydro- or nanogels sensitive to the acidic tumor microenvironment for targeted release of the loaded nanocarrier [46,47]. This local administration could also minimize possible side-effects on monocytic cells targeted from C3 toxin as well as the formation of C3-neutralizing antibodies when applied systemically into the blood stream [29]. In addition, adverse effects of the anti-resorptive activity of (JM173)_3_-Avi-C3 on osteoclasts need to be accessed. Depending on the period of application, it has been shown that the inhibition of osteoclast resorption activity can lead to excessively mineralized bone prone to fragility [48,49]. Furthermore, biomechanical conditions at the site of action need to be considered during local application in the bone niche, e.g., when administered in long bones vs. the vertebral bodies. Nevertheless, there are existing animal models using injections of MDA-MB231 or 4T1 cells to induce breast cancer metastases in mice [50,51]. This would allow us to transfer the in vitro data collected for (JM173)_3_-Avi-C3 in the present study to in vivo experiments.

In summary, the supramolecular bioconjugate (JM173)_3_-Avi-C3 has potential to reduce bone degradation and inhibit BC cell growth and proliferation, making it a promising candidate to break the vicious cycle of osteolysis and tumor growth in the bone niche of late-stage breast cancer patients.

## Data Availability

The original contributions presented in the study are included in the article, further inquiries can be directed to the corresponding author.

## Supplementary Materials

The following supporting information can be downloaded at: https://www.mdpi.com/article/10.3390/jfb16110399/s1. Figure S1: Liquid chromatogram and mass spectrum of B-JM173 after HPLC purification. Figure S2: Synthesis and characterization of B-C3. Figure S3: CXCR4 expression levels in A431, 4T1, MDA-MB231, SupT1, and SupT1ΔCXCR4 cells. Table S1: Scoring system for calculation of overall toxicity in the in vivo zebrafish model.

## Abbreviations

The following abbreviations are used in this manuscript:

4T1	Mouse model cell line of stage IV human breast cancer
A431	Human epidermoid carcinoma cell line
ADP	Adenosine diphosphate
Avi	Avidin
BC	Breast carcinoma
B-C3	Biotinylated C3bot1 toxin
BDP	BODIPY
B-JM173	Biotinylated JM173 peptide
BSA	Bovine serum albumin
C3	C3bot1 toxin from *Clostridium botulinum*
CXCR4	C-X-C chemokine receptor type 4
DMF	Dimethyl formamide
DMSO	Dimethyl sulfoxide
EPI-X4	Endogenous peptide inhibitor for CXCR4 (fragment of human serum albumin, amino acids 408-423)
FCS	Fluorescence correlation spectroscopy
GST	Glutathione-S-transferase
HABA	4’-hydroxyazobenzene-2-carboxylic acid
HEPES	4-(2-hydroxyethyl)-1-piperazineethanesulfonic acid
HPLC	High performance liquid chromatography
JM173	Optimized peptide derived from EPI-X4
LB	Lysogeny broth
LC-MS	Liquid chromatography-mass spectrometry
MALDI-ToF	Matrix-assisted laser desorption/ionization time-of-flight
MDA-MB231	Human mammary gland adenocarcinoma cell line
MS	Mass spectrometry
NAD^+^	Nicotinamide adenine dinucleotide
NC	Negative control
Ns	Not significant
RAW 264.7	Murine macrophage cell line from an Abelson murine leukemia virus-induced tumor
rhM-CSF	Recombinant human macrophage colony-stimulating factor
rhRANKL	Recombinant human receptor activator of nuclear factor kappa-B ligand
Rpm	Rounds per minute
RT	Room temperature
SD	Standard deviation
SupT1	Human T-cell lymphoma cell line
SupT1ΔCXCR4	SupT1 cell line with a CXCR4 knockout
TRAP	Tartrate-resistant acid phosphatase
