# Supplementary material for: Multifunctional Peptide-Based Biohybrid for Targeted Reduction of Metastatic Breast Carcinoma-Associated Osteolysis
Source: J Funct Biomater. 2025 Oct 25;16(11):399. doi: 10.3390/jfb16110399 (PMC12653180; doi:10.3390/jfb16110399)
Supplement: Supplementary file 1 [file jfb-16-00399-s001.zip › jfb-3933151-supplementary.pdf]

Supplements

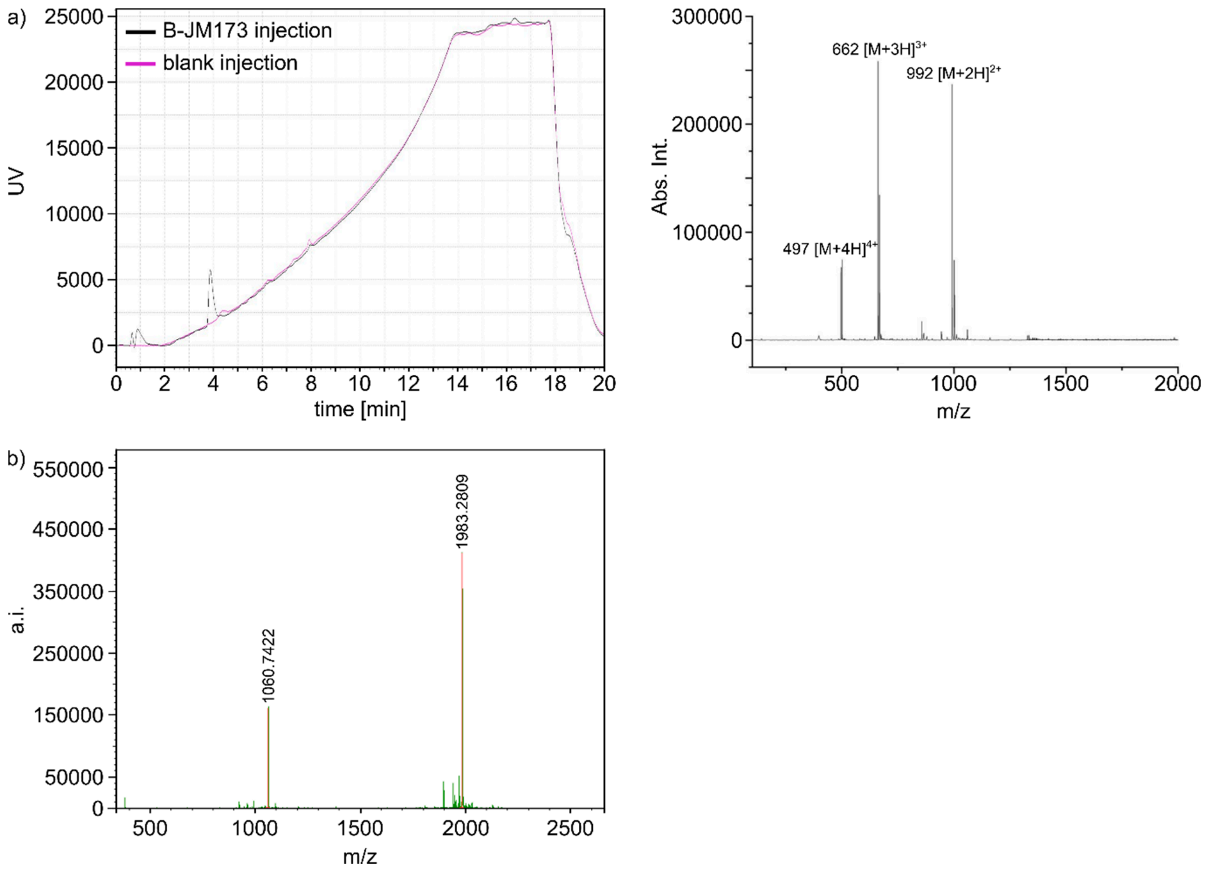

Figure S1: a) Liquid chromatogram of B-JM173 after HPLC purification. Black: B-JM173; pink: blank injection showing background (left) and mass spectrum (right). b) MALDI-ToF mass spectrum of B-JM173

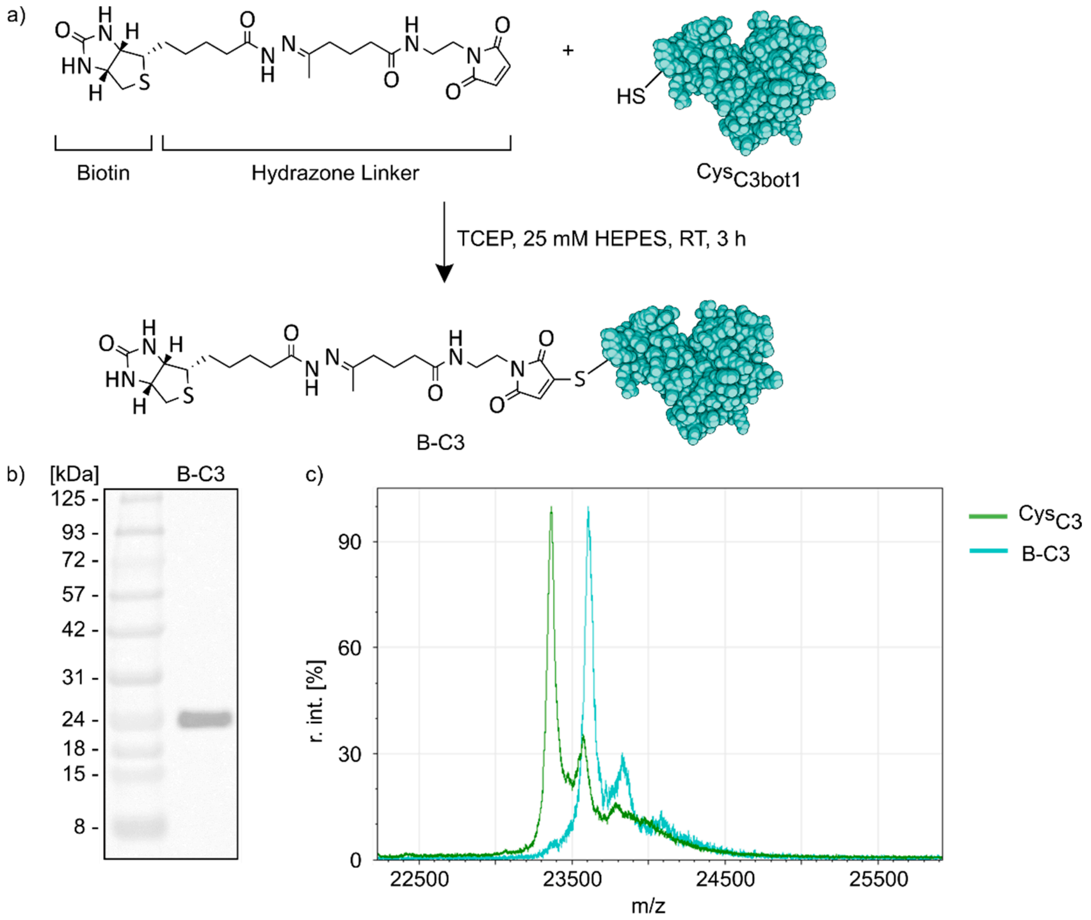

Figure S2: Synthesis and characterization of B-C3. (a) Synthesis of mono-biotinylated C3 toxin (B-C3) with an acid-responsive hydrazone linker. (b) Western Blot analysis of B-C3 detected with Strep-POD for proof of depletion of residual biotin. (c) Matrix-assisted laser desorption/ionization time-of-flight mass spectrometry (MALDI-ToF) analysis of ^{Cys}C3 (green, *m/z* = 23360) and B-C3 (blue, *m/z* = 23618, corresponds to maleimide-ketone due to cleavage of hydrazone bond during sample preparation under acidic condition) using synaptic acid (SA) matrix.

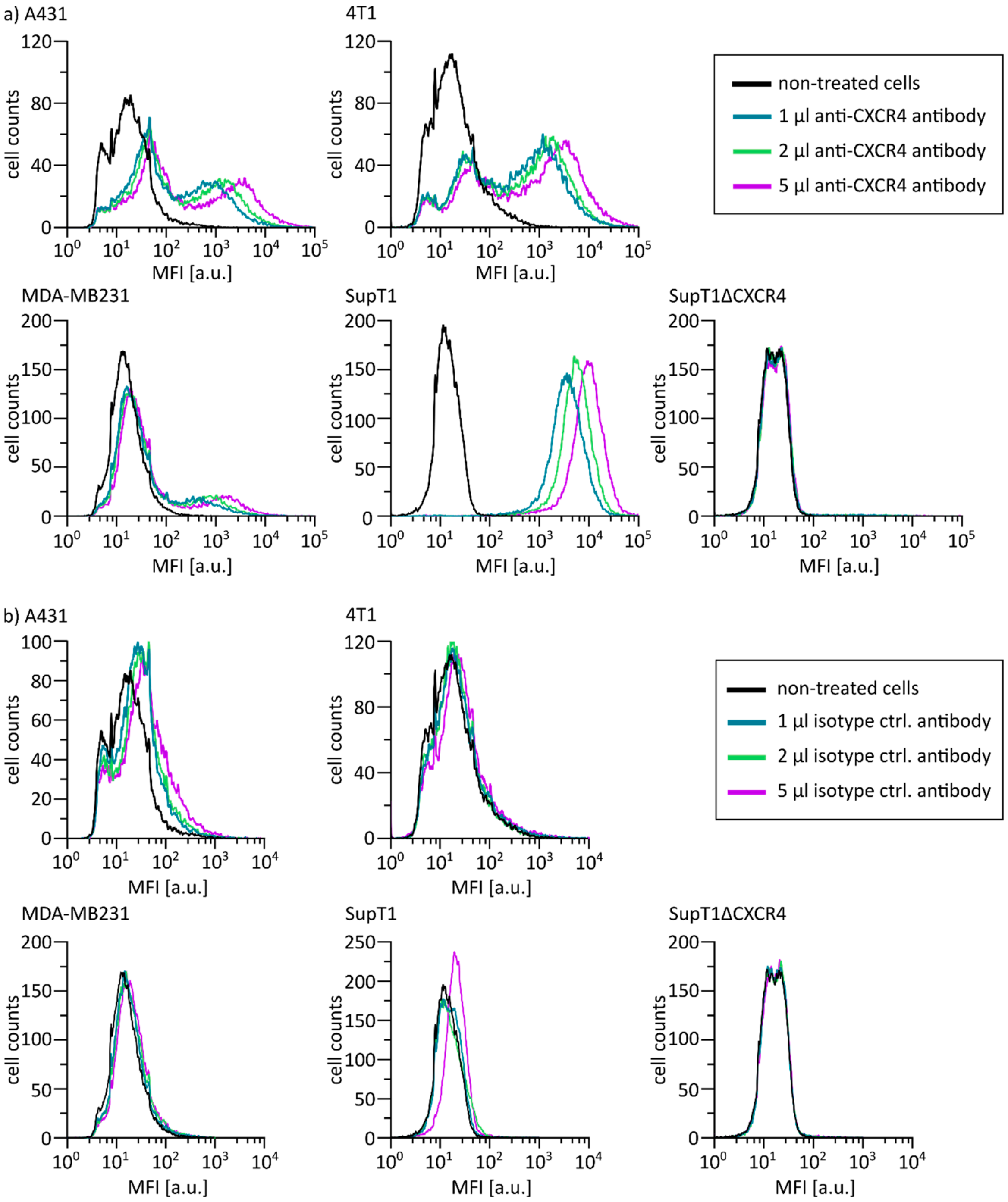

Figure S3: CXCR4 expression levels in A431 cells, 4T1 cells, MDA-MB231 cells, SupT1 cells and SupT1 cells with a CXCR4 knockout (SupT1ΔCXCR4) **(a)** Each cell line was incubated on ice with the indicated volumes of a fluorescently labelled anti-CXCR4 antibody. Antibody binding was detected via flow cytometry and is indicated as a right shift in mean fluorescence intensity (MFI) compared to the non-treated control (black). Data from three independent experiments. **(b)** Treatment of the cell lines with the indicated volumes of a fluorescently labelled rat IgG2a κ isotype control antibody. Detection of binding as described in (a).

Table S1: Scoring system for calculation of overall toxicity in the *in vivo* zebrafish model..

Phenotype	Group	Description
Cytotoxicity	L1	Few lysed cells floating in the medium, embryo looks like untreated control
	L2	Lysed cells in the medium, embryo shows some visible tissue damage
	L3	Embryo shows strong tissue damage
	L4	Embryo is completely disintegrated
	Nec1	Individual necrotic cells
	Nec2	Many necrotic cells
Developmental Toxicity	D1	Developmental delay
	D2	Developmental defect (malformations)
Cardiotoxicity	C1	Reduced circulation or heart edema
	C2	Reduced circulation and heart edema
	C3	No circulation with or w/o heart edema
Neurotoxicity	N0	Normal movement in response to touch
	N1	Reduced movement in response to touch
	N2	No movement in response to touch
Overall classification (combination of phenotypes from above)	wild-type	No visible phenotype and normal movement
	sublethal phenotype	Embryo classified as falling in any of these categories (singly or in combination): L1, L2, Nec1, D1, D2, C1, C2, C3, N1, N2
	lethal phenotype	Embryo classified as falling in any of these categories: Nec2, L3, L4
